# Glycosylation of Receptor Binding Domain of SARS-CoV-2 S-Protein Influences on Binding to Immobilized DNA Aptamers

**DOI:** 10.3390/ijms23010557

**Published:** 2022-01-05

**Authors:** Fedor Grabovenko, Liudmila Nikiforova, Bogdan Yanenko, Andrey Ulitin, Eugene Loktyushov, Timofei Zatsepin, Elena Zavyalova, Maria Zvereva

**Affiliations:** 1 Chemistry Department, Moscow State University, 119991 Moscow, Russia; grabovenko.f@rambler.ru (F.G.); abrludmila@gmail.com (L.N.); tsz@yandex.ru (T.Z.); 2 Biogenec Joint-Stock Company, Moscow Region, 142290 Pushchino, Russia; b.yanenko@biogenec.ru (B.Y.); 1974snail@gmail.com (A.U.); e.loktushov@biogenec.ru (E.L.); 3 Skolkovo Institute of Science and Technology, 121205 Moscow, Russia

**Keywords:** coronavirus, SARS-CoV-2, aptamers, receptor-binding domain, spike glycoprotein, biolayer interferometry, dissociation constants, sensor surface

## Abstract

Nucleic acid aptamers specific to S-protein and its receptor binding domain (RBD) of SARS-CoV-2 (severe acute respiratory syndrome-related coronavirus 2) virions are of high interest as potential inhibitors of viral infection and recognizing elements in biosensors. Development of specific therapy and biosensors is complicated by an emergence of new viral strains bearing amino acid substitutions and probable differences in glycosylation sites. Here, we studied affinity of a set of aptamers to two Wuhan-type RBD of S-protein expressed in Chinese hamster ovary cell line and *Pichia pastoris* that differ in glycosylation patterns. The expression system for the RBD protein has significant effects, both on values of dissociation constants and relative efficacy of the aptamer binding. We propose glycosylation of the RBD as the main force for observed differences. Moreover, affinity of a several aptamers was affected by a site of biotinylation. Thus, the robustness of modified aptamers toward new virus variants should be carefully tested.

## 1. Introduction

The coronavirus disease 2019 (COVID-19) outbreak rapidly spread over the globe and became a pandemic with a huge impact on the world’s social life, healthcare, and economy. More than 100 million infection cases and 3 million deaths have been reported to date. One of the most important issues during the pandemic is the wide application of early and high precision diagnostics. Thus, development of efficient rapid sensing systems for COVID-19 detection is a highly important area of research. The search for simple and robust techniques is an ongoing issue [1,2,3].

The causative pathogen of respiratory pneumonia is a novel beta-coronavirus SARS-CoV-2 (severe acute respiratory syndrome-related coronavirus 2). Spike S-protein plays a key role in the cell entry process of the virus. This is a heavily glycosylated class I homotrimeric fusion protein. Each protein comprises S1 and S2 subunits. S1 subunit includes N-terminal domain and receptor-binding domain (RBD). On the cell surface, S-protein binds specifically to receptor angiotensin-converting enzyme 2 (ACE2) via RBD, which is a necessary step for membrane fusion. Being essential for the SARS-CoV-2 lifecycle, this step makes RBD a promising target for drug and detection systems development. Each S-protein has two prefusion states: ‘RBD-up’ and ‘RBD-down’. The receptor-binding motif of RBD is available only in ‘up’ conformation. The most immunogenic spike epitope is hidden when RBD is down, which apparently helps the virus to evade immune surveillance and causes prolonged recovery [4]. Thus, a potent RBD-blocking agent must bind RBD very tightly and, preferably, have a high association rate as well as low dissociation rate.

Development of efficient, specific treatment is complicated by the emergence of new mutant forms of the virus. Nevertheless, the rate of introduction of new mutations is rather low for coronaviruses compared to influenza viruses, for which the number of different genome variants identified in humans is over a hundred [5]. The World Health Organization currently highlights several lineages as variants of concern due to their high spread among human population globally including delta and omicron variants. Several other lineages are under close attention being a potential threat [6]. These variants of concern have mutations in RBD of S-protein complicating the development of neutralizing therapeutics.

Among different classes of recognizing molecules, nucleic acid aptamers are of particular interest. Aptamers are artificial molecules derived from huge libraries of random oligonucleotides during a procedure called SELEX (Systematic Evolution of Ligands by EXponential Enrichment). Aptamers recognize a part of the protein surface with high affinity and specificity. Aptamers compete with antibody-based approaches due to the capability of large-scale chemical synthesis, low toxicity and immunogenicity, and simplicity of introduction of almost any site-specific chemical modification. Several studies on DNA and XNA (xeno nucleic acids that contains artificial nucleotides) aptamer selection to SARS-CoV-2 proteins have been published [7,8,9,10,11,12,13,14,15]. Most aptamers target the RBD of S-protein due to steric availability among other SARS-CoV-2 proteins [7,9,10,13,14,15]. Some of the aptamers were shown to inhibit RBD interaction with the ACE2 [7,10,13,14,15] and viral infection of mammalian cell lines [7,14].

The aptamers have been successfully applied for the detection of S-protein [10,16], S-protein-functionalized pseudoviruses [9,10,17], or SARS-CoV-2 virus [11,12,18]. Aptamer specificity is a key feature that affect robustness of aptamer-based aptasensors. Aptamers with similar affinities to different SARS-CoV-2 variants are of interest to develop a united test system. Aptamer MSA1 binds S-protein from UK lineage with 10-fold lower dissociation constant (K_D_) than S-protein from the original Wuhan strain, whereas MSA5 does not discriminate the proteins [9]. Dimeric aptamer DSA1N5 has 2-4-fold differences in K_D_ for different variants: Wuhan, UK, and Indian ones were compared [17]. Similarly, aptamer FANA-R8-9 binds S-protein from Delta variant with 10-fold lower K_D_ than S-protein from the original Wuhan strain [15].

One of the issues facing the developers of aptasensors is understanding the effect of fixing on the surface of aptamers without losing effective interaction with the target. The aptamers development during selection includes formation of a stable secondary structure, but the ends may be also needed for target recognition, which will lead to a loss of sensor efficiency upon immobilization. It is also necessary to consider the possible influence of posttranslational modifications of the protein on the recognition by the aptamers.

In our study, we compared affinity of aptamers derived by Song et al. [13] to two Wuhan-type RBD-proteins expressed in Chinese hamster ovary (CHO) cell line and *Pichia pastoris* that differ in post-translational glycosylation. The expression system for the RBD protein significantly affects both K_D_ values and relative efficiency of the aptamers. Therefore, we propose that glycosylation is a main force of the observed differences. As new virus variants can differ in glycosylation, robustness of aptamers should be carefully tested for all new variants.

## 2. Results and Discussion

We tested two recombinant RBD proteins with the same amino acid sequence and different glycosylation. One was produced in a CHO cell line (a mammalian cell line), another one in *Pichia pastoris* (yeasts). The first stage of post-translational protein modification is similar for both organisms and results in addition of Man_8_GlcNAc_2_ (Man—mannose, GlcNAc—N-acetylglucosamine) to Asn and Gln. In mammalian cells this oligosaccharide then undergoes transformation into Sia_2_Gal_2_GlcNAc_2_Man_3_GlcNAc_2_ (Sia—sialic acid, Gal—galactose), while in yeast, Man_8_GlcNAc_2_ is expanded with mannose residues yielding Man_15-30_GlcNAc_2_ clusters [19]. In this study we used an RBD protein with homogeneous glycosylation in CHO cell line as judged by denaturating polyacrylamide gel electrophoresis (PAGE) that provided a molecular weight about ~37 kDa [20]. Glycosylation of RBD in *P. pastoris* proceeded heterogeneously; ~2/3 of the protein had molecular weight ~23 kDa, and ~1/3 had molecular weight close to 40 kDa (Figure S1).

**Figure 1:**
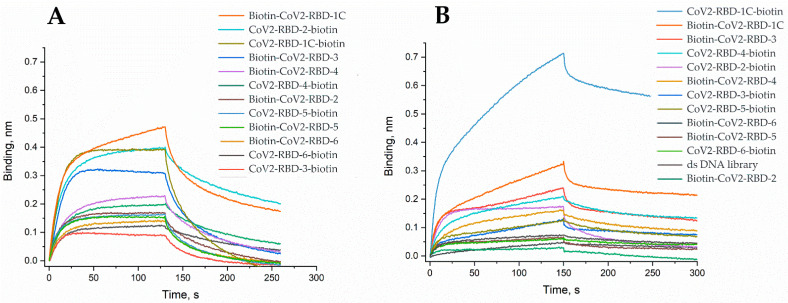
Binding curves of immobilized biotinylated aptamers to RBD proteins expressed in Chinese hamster ovary cell line (**A**) and *Pichia pastoris* (**B**). The concentration of RBD proteins was 100 nM.

Six DNA aptamers developed by Song et al. [13] were modified with biotin residue either at 5′- or 3′-end. Affinity experiments were performed for aptamers bearing 3′- or 5′-biotin using both RBD variants. A biotinylated aptamer was immobilized onto streptavidin sensor chip, and binding of soluble RBD protein was studied using bio-layer interferometry (BLI) approach (Table 1, Figure 1).

Glycosylation of the protein affected K_D_ for almost all aptamers. Yeast protein gives 4–29-fold lower K_D_ for the same aptamer sequence and biotinylation site (Table 1). In this case, presence or absence of glycosylation destabilizes most of the aptamer–protein complexes. Interestingly, among our set, two aptamers have nearly the same affinity to both proteins (CoV2-RBD-2-Biotin and Biotin-CoV2-RBD-4), whereas two other aptamers bind CHO-derived protein only (Biotin-CoV2-RBD-2 and Biotin-CoV2-RBD-5) with almost no affinity to the RBD from yeast. The most probable reason for these differences is a high content of poorly glycosylated or over-glycosylated RBD expressed in *P. pastoris*.

These experimental data are in full agreement with the structures of aptamer–S-protein complex predicted by molecular dynamic simulation [21]. The sites of CoV2-RBD-1C binding were predicted to be different in glycosylated protein and non-glycosylated protein, whereas CoV2-RBD-4 aptamer binds both forms in the same manner [19]. Our data revealed 7–18-fold differences in K_D_ for CoV2-RBD-1C and only 1.3–3.8-fold differences in K_D_ for CoV2-RBD-4 (Table 1).

Reconsideration of the dataset, including the maximal signal at the plateau at the association step, allows indirect estimation of fullness of the biolayer. A half of the aptamers have 2–4-fold lower layer occupancy (below the line in Table 1) that could be interpreted as a result of a mix of active and inactive aptamer conformations or, alternatively, a mix of recognizable and unrecognizable RBD conformations. In most cases, binding of both types of RBD protein is low for the aptamers below the line (Table 1). Thus, suboptimal aptamer conformation is the most probable reason of the partial fullness of the biolayer. Six most efficient aptamers comprise both glycosylation-tolerant molecules (aptamers CoV2-RBD-2-Biotin, Biotin-CoV2-RBD-4, CoV2-RBD-4-Biotin) and aptamers with high affinity to nonglycosylated form (aptamers Biotin-CoV2-RBD-1C, CoV2-RBD-1C-Biotin and Biotin-CoV2-RBD-3) with K_D_ ratio in the range of 4-29 nM.

Differences in K_D_ result mainly from varied dissociation kinetic constants (Table 2). Thus, glycosylation affects the stability of aptamer-RBD complexes. Additional deglycosylation procedure during sample preparation could be proposed to achieve the highest sensitivity in detection of SARS-CoV-2 viruses.

This dataset revealed aptamer CoV2-RBD-2-Biotin as one more promising recognition element as it had similar affinity to proteins with different glycosylation, as well as similar signal intensity at the plateau which indicates similar fullness of the sensor surface. On the contrary, the same aptamer with other site of biotinylation, Biotin-CoV2-RBD-2, binds CHO-derived protein only. Thus, the site of modification is crucial for robustness of this aptamer.

## 3. Materials and Methods

### 3.1. Production of a Recombinant RBD in P. pastoris and Purification

Based on the published data we have selected the RBD sequence (accession number YP_009724390.1) consisting of residues 319 to 541. The RBD sequence was codon optimized for *P. pastoris* expression and cloned into pD912 vector (Atum, Newark, CA, USA) with AOX1 promoter and a full MAT alpha signal sequence for efficient secretion. The construct was designated with an additional eight His residues at the C-end to perform Ni-metal affinity purification. pRBD vector (~20 µg) was linearized with the SacI enzyme (NEB, Ipswich, MA, USA) and purified by miniprep kit (Eurogen, Moscow, Russia). Linearized plasmid (~1 µg) was transformed into *P. pastoris* BG-11 strain (Atum, Newark, CA, USA) by electroporation according to the protocol of Thermo Fisher. Transformants were selected on YPD plates at a zeocin concentration (Thermo Fisher Scientific, Waltham, MA, USA) of 500 µg/mL after incubation for 3 days at 30 °C. Ten colonies from the YPD plate were picked and screened for expression by inducing with 1% methanol that was added every 24 h. Tubes (15 mL) containing 1 mL YPD media were used for growing the cultures for up to 120 h maintained at 30 °C and 250 rpm. The expression levels were monitored by dot blot analysis with anti-RBD antibodies (Chema, Moscow, Russia). The colony showing the highest expression level was then chosen for large-scale expression. Larger-scale cultivation was performed in shake flasks by maintaining the same volumetric ratio (flask: media) as the small-scale cultures. Cultures were harvested by centrifuging at 4000× *g* and a subsequent filtering through a 0.45 μm filter. The supernatant was loaded to pre-equilibrated Ni Sepharose (Cytiva, Marlborough, MA, USA) and washed with 1× PBS (pH 7.4) containing 20 mM imidazole. RBD protein was eluted in 1× PBS (pH 7.4) containing 300 mM imidazole or 25 mM EDTA. The eluted fractions with the protein (SDS-PAGE control) were pooled and dialyzed against 1× PBS (pH 7.4) to remove imidazole.

### 3.2. SDS-PAGE and Western Blot Analysis

Common SDS-PAGE (PAGE with sodium dodecyl sulphate) was used to estimate the protein purity. The acrylamide concentration was 5% in the concentration gel and 10% in separation gel. For western blotting, proteins were transferred onto membrane followed by blocking with fat-free milk. The membrane was washed with PBST (1× PBS with 0.05% Tween-20), incubated with antibodies 1:100 (Chema XR06, Moscow, Russia), washed, and incubated with anti-mouse AP conjugated antibody (Chema, Moscow, Russia) at 1:1000. Blot was visualized using DAB substrate (Sigma, New York, NY, USA). Both bands from denaturating electrophoresis of RBD protein from *P. pastoris* (Figure S1) were stained by specific antibodies (Figure S2).

### 3.3. Oligonucleotides

Oligonucleotides were synthesized using commercially available reagents by a solid-phase phosphoramidite method, followed by high performance liquid chromatography (HPLC) purification. The sequences and sites of biotinylation are provided in Table 3.

To provide proper folding, aptamer stock solution (2 μM) in PBS buffer with 0.55 mM MgCl_2_ was preheated at 95 °C for 5 min, then cooled down to 0 °C on ice for 5 min and, finally, was kept at room temperature for 10 min.

### 3.4. Binding Experiments

The stock solution of the aptamer was diluted with 5X binding buffer and distilled water to obtain the final concentration of 200 nM aptamer in 1X binding buffer (PBS buffer, 0.55 mM MgCl_2_, 0.1 mg/mL BSA, 0.002% Tween-20). To test the binding specificity, the experiments with biotinylated 71 bp dsDNA with randomized sequence were conducted (5′-CTCCTCTGACTGTAACCACG-N-N1-N-N1-N-N1-N-N1-N2-N2-N2-N2-N-N1-N-N1-N-N1-N-N1-N-N1-N2-N2-N2-N-N1-N-N1-N-N1-GCATAGGTAGTCCAGAAGCC-3′, where N = 45:05:45:05 A/C/G/T; N1 = 05:45:05:45 A/C/G/T; N2 = 25:25:25:25 A/C/G/T). The library was used as a reference for aptamers. All the experiments were carried out at room temperature.

Biolayer interferometry assays were performed on a BLItz (ForteBio, Fremont, CA, USA) instrument at advanced kinetics mode with shaking at 2200 RPM. Streptavidin biosensors (ForteBio, Fremont, CA, USA) were hydrated in binding buffer for 10 min prior to the experiment. The optimized BLI protocol comprised the following steps:

1. Initial baseline carried in binding buffer for 90 s;
2. Loading of aptamer for 210 s;
3. The second baseline in binding buffer for 30 s;
4. Association for 150 s (50, 100 or 200 nM protein solution in binding buffer);
5. Dissociation in binding buffer for 150 s;
6. Washing step-1 carried in 1 M ethanolamine (pH 8.3) for 120 s;
7. Washing step-2 carried in binding buffer for 120 s.

Each measurement step was carried out in black tubes (Sigma-Aldrich, New York, NY, USA) with at least 300 μL of a respective solution in three repeats.

Binding curves are provided in the Supplementary Materials (Figures S3–S26). The curves were approximated exponentially using 1:1 binding model according to the guidelines [22].

## 4. Conclusions

Two RBD proteins with the same amino acid sequence, but different glycosylation patterns can be distinguished by DNA aptamers. Glycosylation of the RBD protein has significant effects, both on values of dissociation constants and relative efficacy of the aptamer binding. Moreover, the affinity of a several aptamers was affected by the site of biotinylation. Thus, optimization of modified aptamers toward new virus variants of SARS-CoV-2 virus should be carefully performed.

## Figures and Tables

**Table 1:** Estimated dissociation constants for aptamer-RBD complexes and maximal signal of the binding curves in 100 nM solution of RBD protein. Aptamers are ranged according to maximal signal of the binding curves for RBD protein expressed in CHO cell line. n.d.—not determinable due to low signal.

Aptamer	RBD Expressed in CHO Cell Line		RBD Expressed in *P. pastoris*	
	K_D_, nM	Max Signal	K_D_, nM	Max Signal
Biotin-CoV2-RBD-1C	5.8 ± 1.4	0.47	0.80 ± 0.16	0.32
CoV2-RBD-2-Biotin	4.3 ± 1.4	0.40	10.0 ± 1.8	0.17
CoV2-RBD-1C-Biotin	27 ± 5	0.39	1.5 ± 0.3	0.71
Biotin-CoV2-RBD-3	40 ± 6	0.32	1.4 ± 0.4	0.23
Biotin-CoV2-RBD-4	18 ± 2	0.22	14 ± 4	0.16
CoV2-RBD-4-Biotin	21 ± 4	0.20	5.5 ± 0.9	0.21
Biotin-CoV2-RBD-2	17 ± 5	0.17	n.d.	0.03
CoV2-RBD-5-Biotin	26 ± 5	0.16	1.20 ± 0.11	0.12
Biotin-CoV2-RBD-5	22 ± 5	0.15	n.d.	0.06
Biotin-CoV2-RBD-6	29 ± 8	0.14	3.5 ± 0.9	0.07
CoV2-RBD-6-Biotin	10 ± 2	0.12	1.0 ± 0.3	0.06
CoV2-RBD-3-Biotin	24 ± 3	0.10	2.0 ± 0.7	0.12

**Table 2:** Estimated association kinetic constants (k_on_) and dissociation kinetic constants (k_off_) for aptamer–RBD complexes. Aptamers are ranged according to maximal signal of the binding curves for RBD protein expressed in CHO cell line. n.d.—not determinable.

Aptamer	RBD Expressed in CHO Cell Line		RBD Expressed in *P. pastoris*	
	k_on_, µM^−1^s^−1^	k_off_, ms^−1^	k_on_, µM^−1^s^−1^	k_off_, ms^−1^
Biotin-CoV2-RBD-1C	1.1 ± 0.3	6.3 ± 1.4	1.5 ± 0.3	1.2 ± 0.4
CoV2-RBD-2-Biotin	0.8 ± 0.2	3.6 ± 1.4	1.0 ± 0.2	10.0 ± 1.2
CoV2-RBD-1C-Biotin	0.73 ± 0.14	19 ± 3	0.9 ± 0.2	1.4 ± 0.3
Biotin-CoV2-RBD-3	0.51 ± 0.08	21 ± 3	1.2 ± 0.3	1.6 ± 0.5
Biotin-CoV2-RBD-4	0.75 ± 0.12	13.9 ± 0.9	0.59 ± 0.15	8 ± 3
CoV2-RBD-4-Biotin	0.53 ± 0.14	11.0 ± 1.6	0.80 ± 0.16	4.3 ± 0.6
Biotin-CoV2-RBD-2	0.8 ± 0.3	14.4 ± 0.8	n.d.	n.d.
CoV2-RBD-5-Biotin	0.8 ± 0.2	20.8 ± 0.9	1.62 ± 0.09	1.9 ± 0.2
Biotin-CoV2-RBD-5	1.1 ± 0.3	24 ± 3	n.d.	n.d.
Biotin-CoV2-RBD-6	0.8 ± 0.2	23.4 ± 0.8	1.7 ± 0.4	5.8 ± 1.6
CoV2-RBD-6-Biotin	0.74 ± 0.13	7.7 ± 1.7	2.0 ± 0.5	2.0 ± 0.8
CoV2-RBD-3-Biotin	0.98 ± 0.12	24 ± 4	0.7 ± 0.3	1.3 ± 0.3

**Table 3:** Sequences and sites of biotinylating od DNA aptamers studied in this work.

Aptamer	Sequence
Biotin-CoV2-RBD-1C	biotin-5′-T_10_-CAGCACCGACCTTGTGCTTTGGGAGTGCTGGTCC-AAGGGCGTTAATGGACA-3′
CoV2-RBD-1C-Biotin	5′-CAGCACCGACCTTGTGCTTTGGGAGTGCTGGTCC-AAGGGCGTTAATGGACA-T_10_-3′-biotin
Biotin-CoV2-RBD-2	biotin-5′-T_10_-ATCCAGAGTGACGCAGCATCGAGTGGTGGGCTGGTC-GGGTTTGGATTCCCTTAGATGCTGGACACGGTGGCTTAGT-3′
CoV2-RBD-2-Biotin	5′-ATCCAGAGTGACGCAGCATCGAGTGGTGGGCTGGTC-GGGTTTGGATTCCCTTAGATGCTGGACACGGTGGCTTAGT-T_10_-3′-biotin
Biotin-CoV2-RBD-3	biotin-5′-T_10_-ATCCAGAGTGACGCAGCACTGCGTAGGCGCGGCCAAT-GTGTAGGATTGCTCAGGTCTGCTGGACACGGTGGCTTAGT-3′
CoV2-RBD-3-Biotin	5′-ATCCAGAGTGACGCAGCACTGCGTAGGCGCGGCCAAT-GTGTAGGATTGCTCAGGTCTGCTGGACACGGTGGCTTAGT-T_10_-3′-biotin
Biotin-CoV2-RBD-4	biotin-5′-T_10_-ATCCAGAGTGACGCAGCATTTCATCGGGTCCAAAA-GGGGCTGCTCGGGATTGCGGATATGGACACGT-3′
CoV2-RBD-4-Biotin	5′-ATCCAGAGTGACGCAGCATTTCATCGGGTCCAAAA-GGGGCTGCTCGGGATTGCGGATATGGACACGT-T_10_-3′-biotin
Biotin-CoV2-RBD-5	biotin-5′-T_10_-ATCCAGAGTGACGCAGCAGGACTGCTTAGGATTGCGAAGCTGAGGAGCTCCCCCGCCTTGGACACGGTGGCTTAGT-3′
CoV2-RBD-5-Biotin	5′-ATCCAGAGTGACGCAGCAGGACTGCTTAGGATTGCGAA-GCTGAGGAGCTCCCCCGCCTTGGACACGGTGGCTTAGT-T_10_-3′-biotin
Biotin-CoV2-RBD-6	biotin-5′-T_10_-ATCCAGAGTGACGCAGCAGTAGGGGGATTGGCTCCAGGG-CCTGGCTGACGGTTGCACGTGGACACGGTGGCTTAGT-3′
CoV2-RBD-6-Biotin	5′-ATCCAGAGTGACGCAGCAGTAGGGGGATTGGCTCCAGGG-CCTGGCTGACGGTTGCACGTGGACACGGTGGCTTAGT-T_10_-3′-biotin

## Data Availability

Not applicable.

## Supplementary Materials

The following are available online at https://www.mdpi.com/article/10.3390/ijms23010557/s1.
