# Supplementary material for: Glycosylation of Receptor Binding Domain of SARS-CoV-2 S-Protein Influences on Binding to Immobilized DNA Aptamers
Source: Int J Mol Sci. 2022 Jan 5;23(1):557. doi: 10.3390/ijms23010557 (PMC8745424; doi:10.3390/ijms23010557)
Supplement: Supplementary file 1 [file ijms-23-00557-s001.zip › ijms-1523739-supplementary.pdf]

Supplementary materials

Glycosylation of Receptor Binding Domain of SARS-CoV-2 S-Protein Influences on Binding to Immobilized DNA Aptamers

Fedor Grabovenko, Liudmila Nikiforova, Bogdan Yanenko, Andrey Ulitin[2], Evgeniy Loktyushov, Timofei Zatsepin, Elena Zavyalova and Maria Zvereva

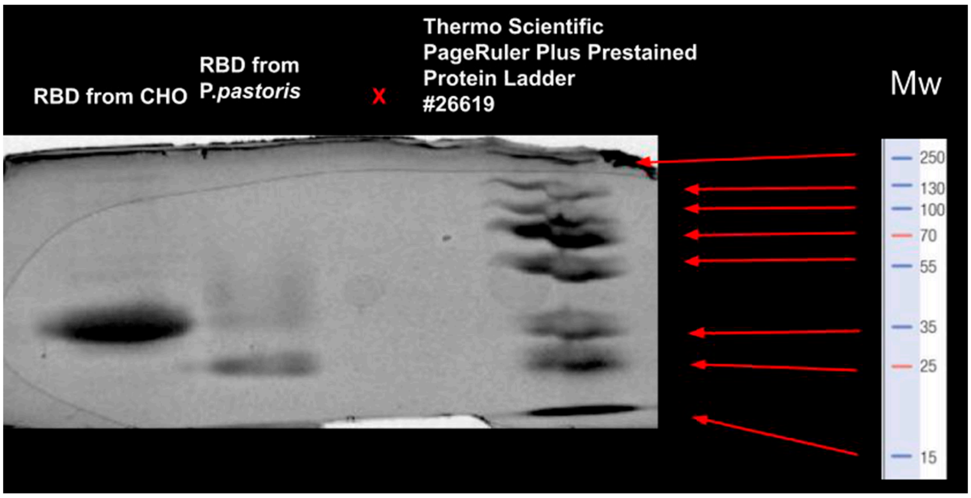

Figure S1. Denaturating polyacrylamide electrophoresis of RBD proteins from CHO cell line and *P. pastoris*. Unprocessed protein runs at 25 kDa, glycosylated proteins run at 35-45 kDa.

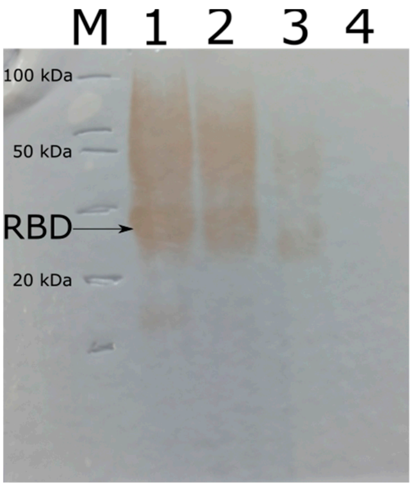

Figure S2. Western blot analysis of RBD protein *P. pastoris*. The track M – molecular weight ladder transfered from polyacrylamide gel; tracks 1-3 RBD protein from *P. pastoris* in different concentrations (1>2>3). The track 4 – BSA.

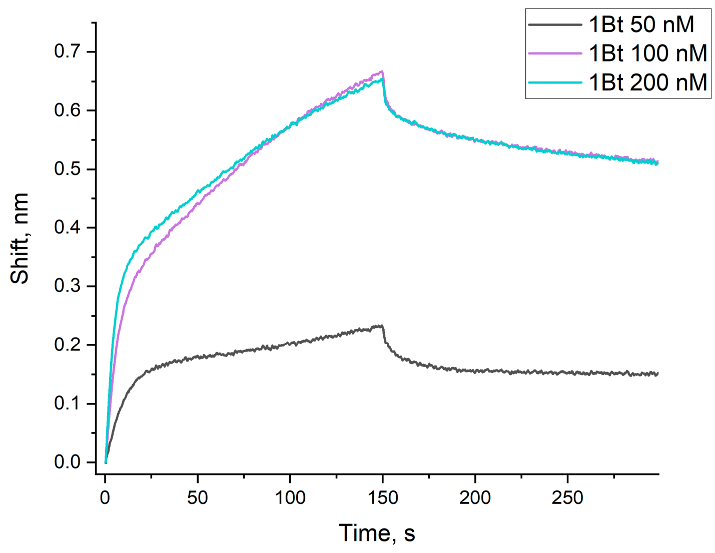

Figure S3. Binding curves of RBD expressed in *P. pastoris* to immobilized aptamer CoV2-RBD-1C-Biotin.

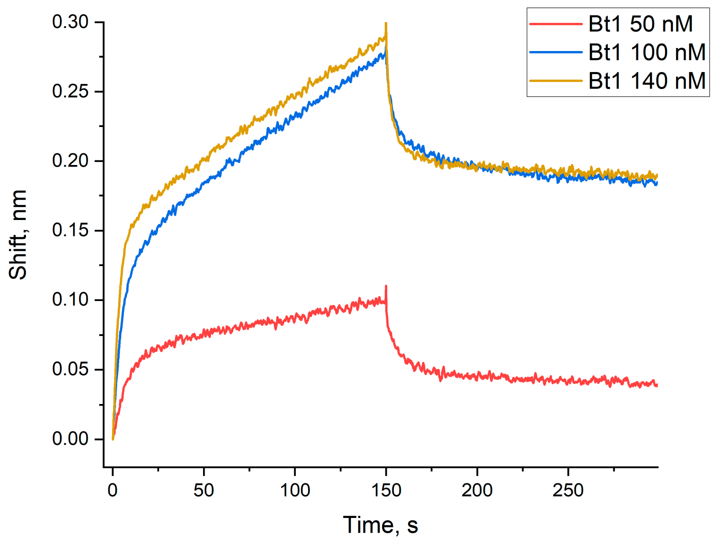

Figure S4. Binding curves of RBD expressed in *P. pastoris* to immobilized aptamer Biotin-CoV2-RBD-1C.

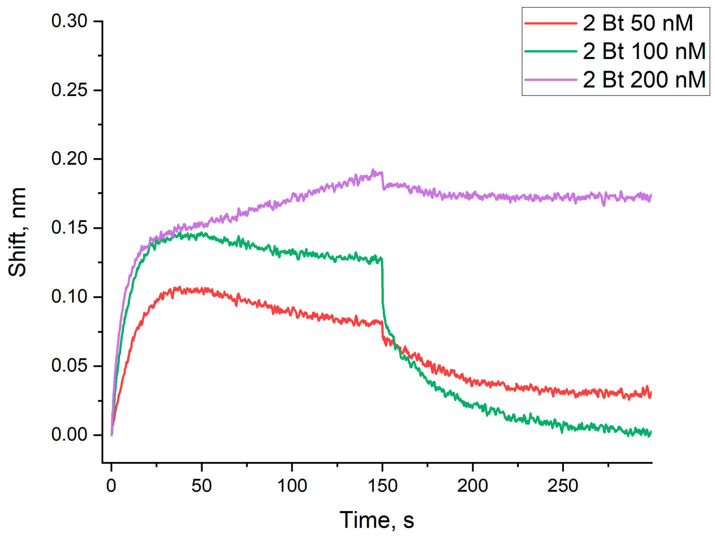

Figure S5. Binding curves of RBD expressed in *P. pastoris* to immobilized aptamer CoV2-RBD-2-Biotin.

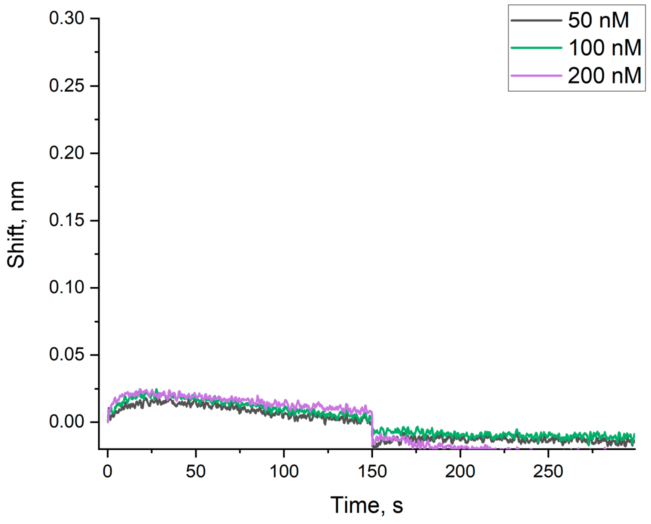

Figure S6. Binding curves of RBD expressed in *P. pastoris* to immobilized aptamer Biotin-CoV2-RBD-2.

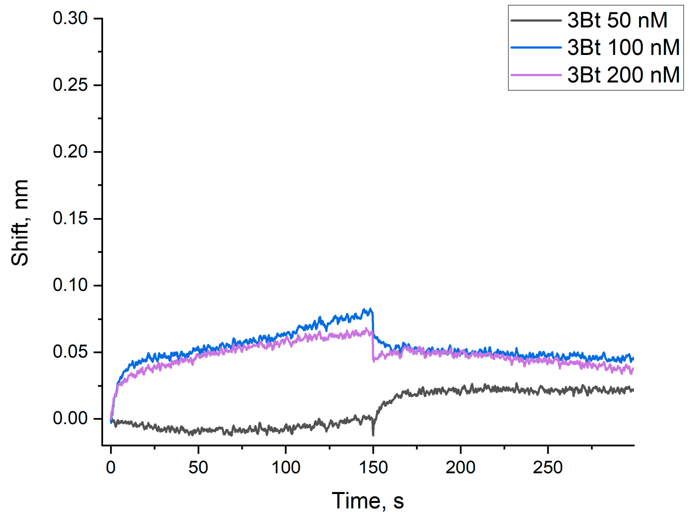

Figure S7. Binding curves of RBD expressed in *P. pastoris* to immobilized aptamer CoV2-RBD-3-Biotin.

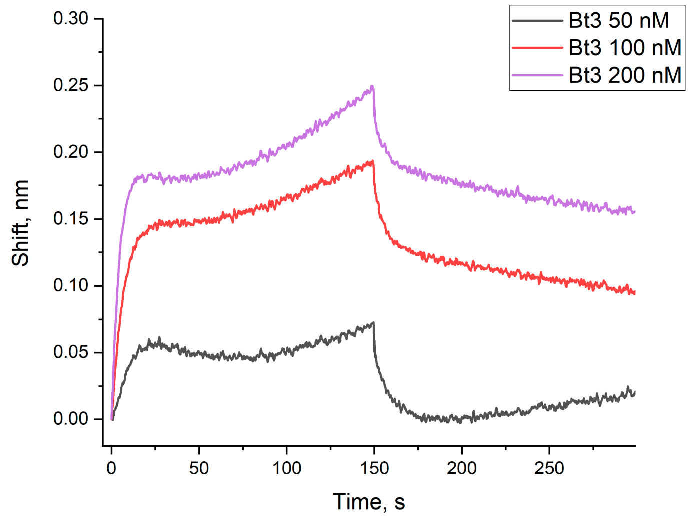

Figure S8. Binding curves of RBD expressed in *P. pastoris* to immobilized aptamer Biotin-CoV2-RBD-3.

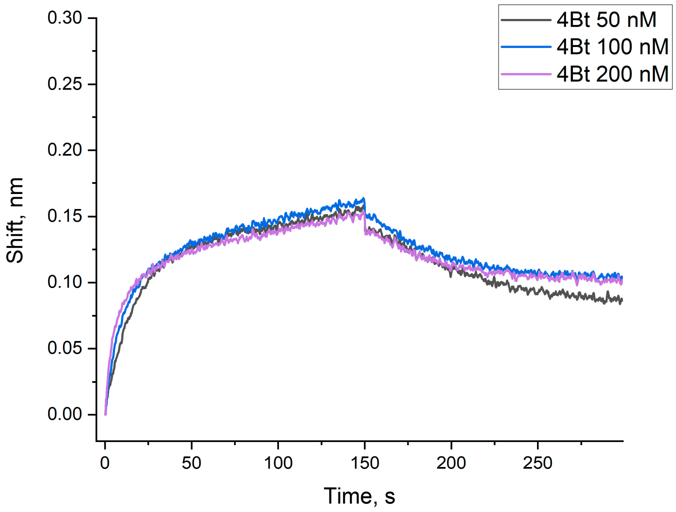

Figure S9. Binding curves of RBD expressed in *P. pastoris* to immobilized aptamer CoV2-RBD-4-Biotin.

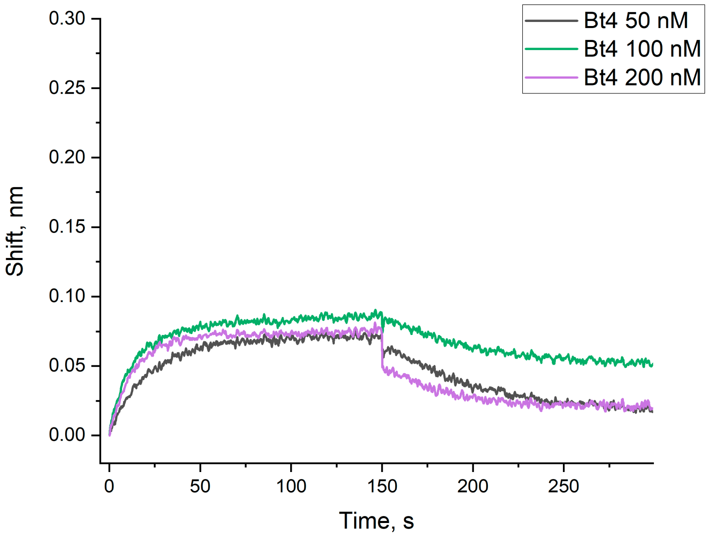

Figure S10. Binding curves of RBD expressed in *P. pastoris* to immobilized aptamer Biotin-CoV2-RBD-4.

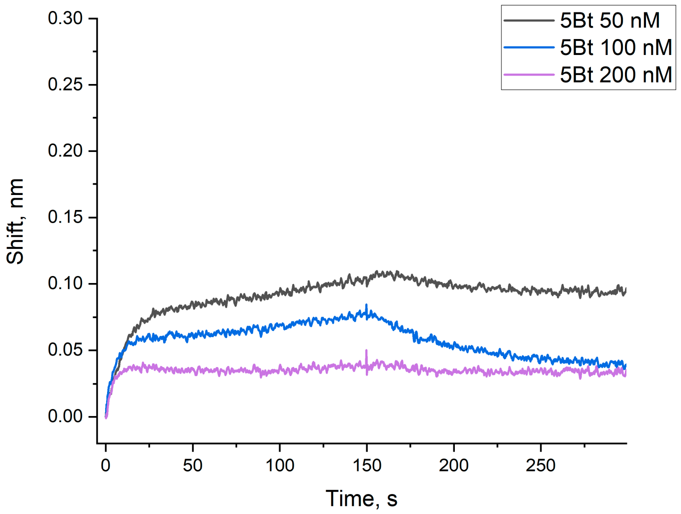

Figure S11. Binding curves of RBD expressed in *P. pastoris* to immobilized aptamer CoV2-RBD-5-Biotin.

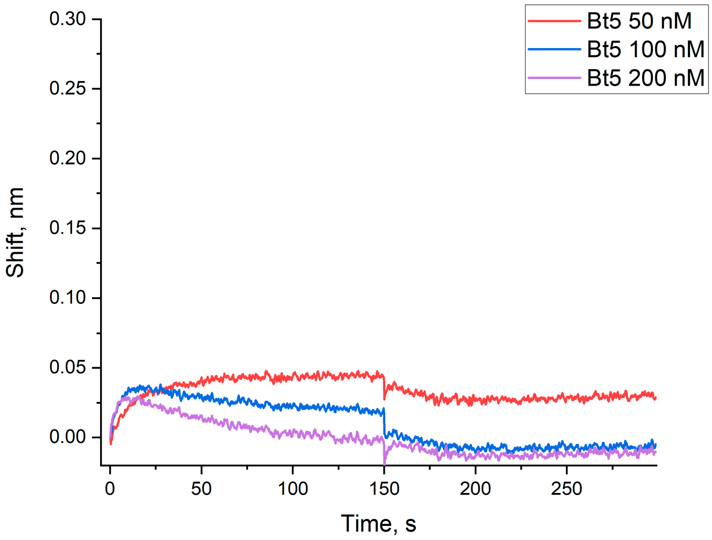

Figure S12. Binding curves of RBD expressed in *P. pastoris* to immobilized aptamer Biotin-CoV2-RBD-5.

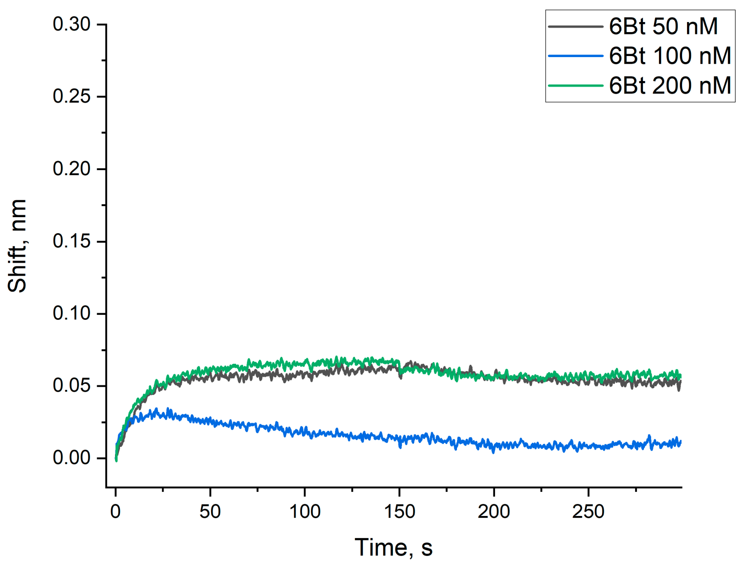

Figure S13. Binding curves of RBD expressed in *P. pastoris* to immobilized aptamer CoV2-RBD-6-Biotin.

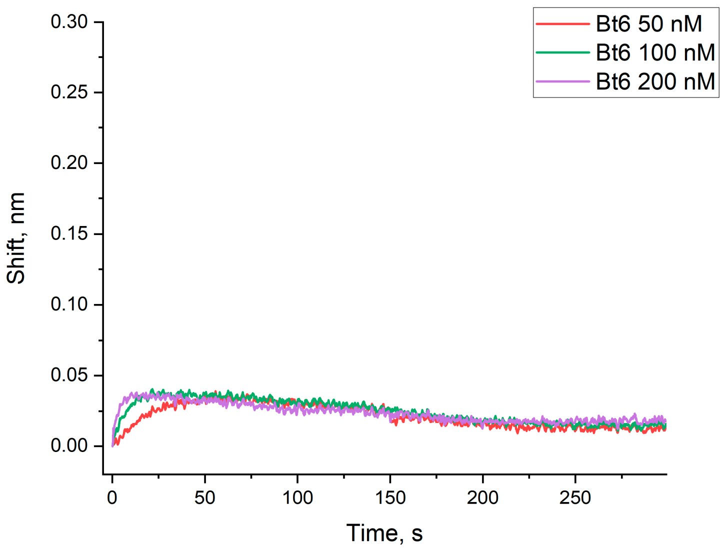

Figure S14. Binding curves of RBD expressed in *P. pastoris* to immobilized aptamer Biotin-CoV2-RBD-6.

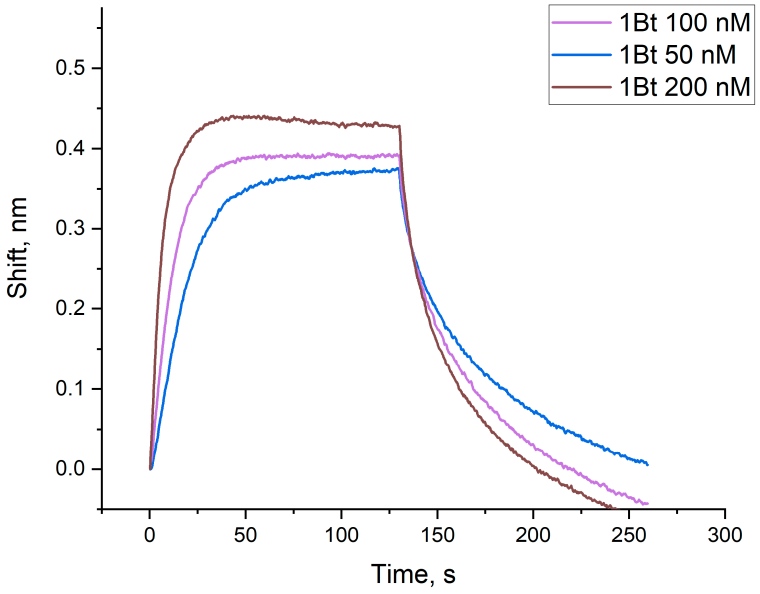

Figure S15. Binding curves of RBD expressed in CHO cell line to immobilized aptamer CoV2-RBD-1C-Biotin.

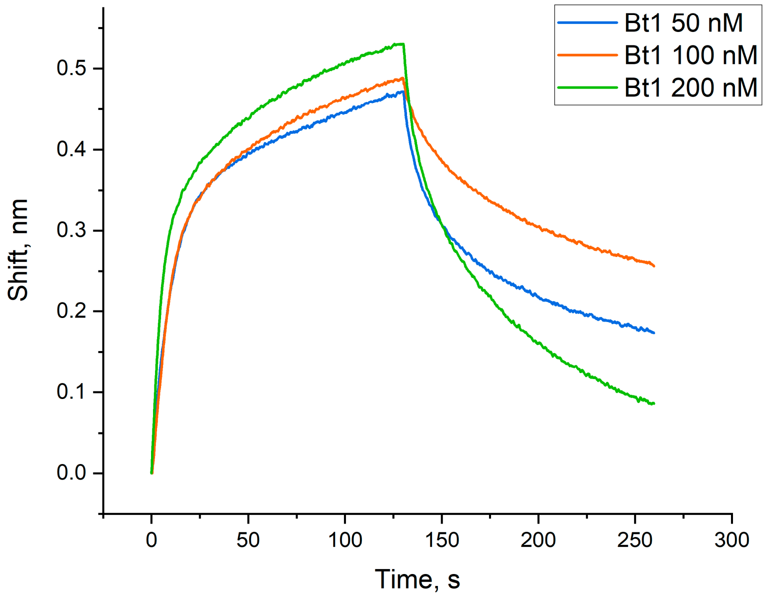

Figure S16. Binding curves of RBD expressed in CHO cell line to immobilized aptamer Biotin-CoV2-RBD-1C.

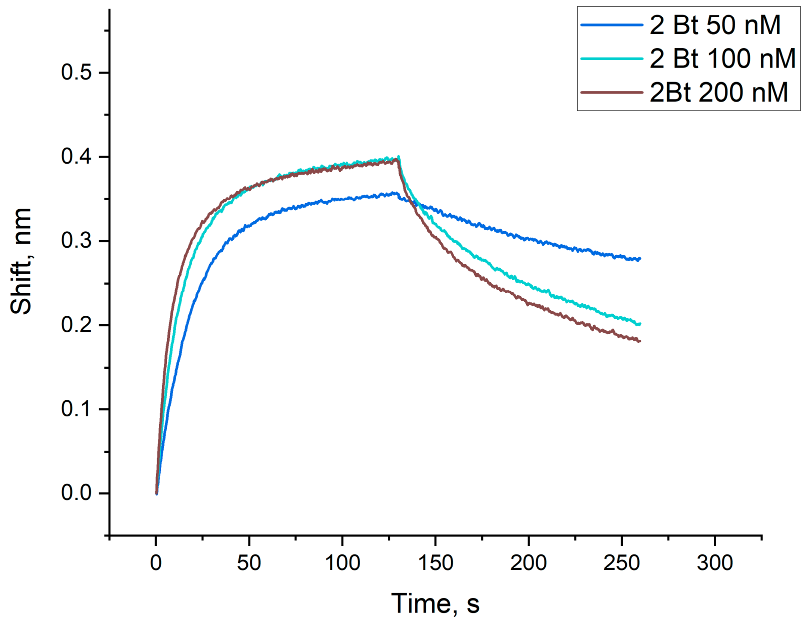

Figure S17. Binding curves of RBD expressed in CHO cell line to immobilized aptamer CoV2-RBD-2-Biotin.

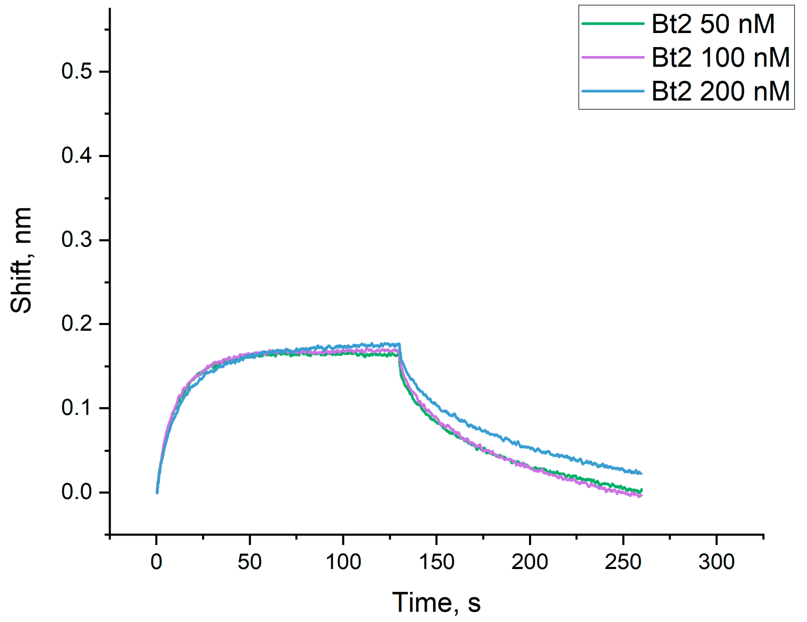

Figure S18. Binding curves of RBD expressed in CHO cell line to immobilized aptamer Biotin-CoV2-RBD-2.

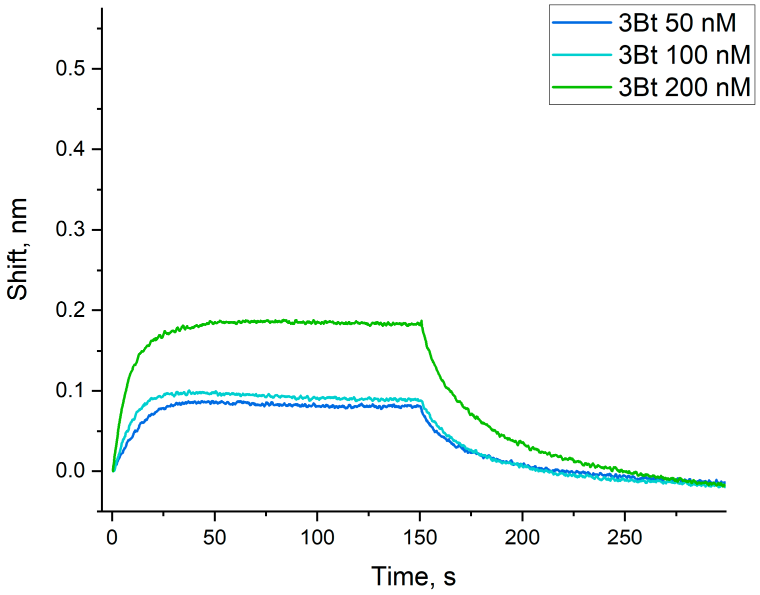

Figure S19. Binding curves of RBD expressed in CHO cell line to immobilized aptamer CoV2-RBD-3-Biotin.

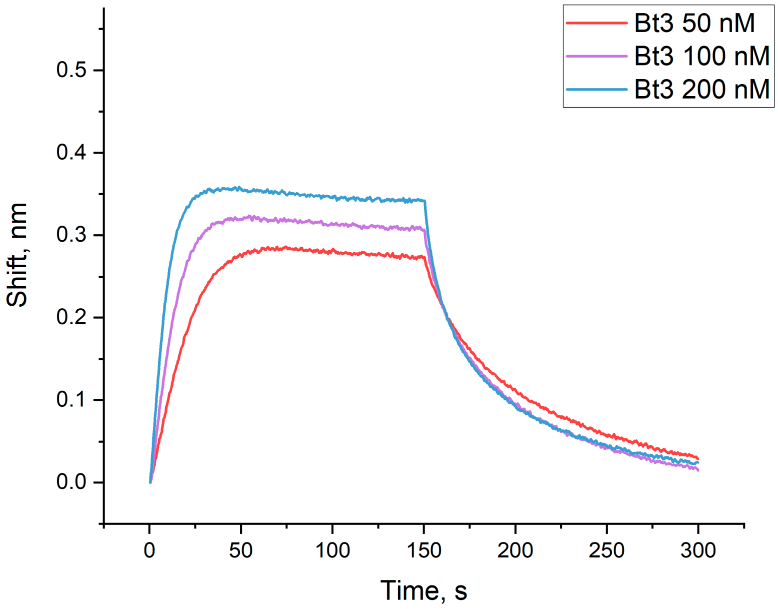

Figure S20. Binding curves of RBD expressed in CHO cell line to immobilized aptamer Biotin-CoV2-RBD-3.

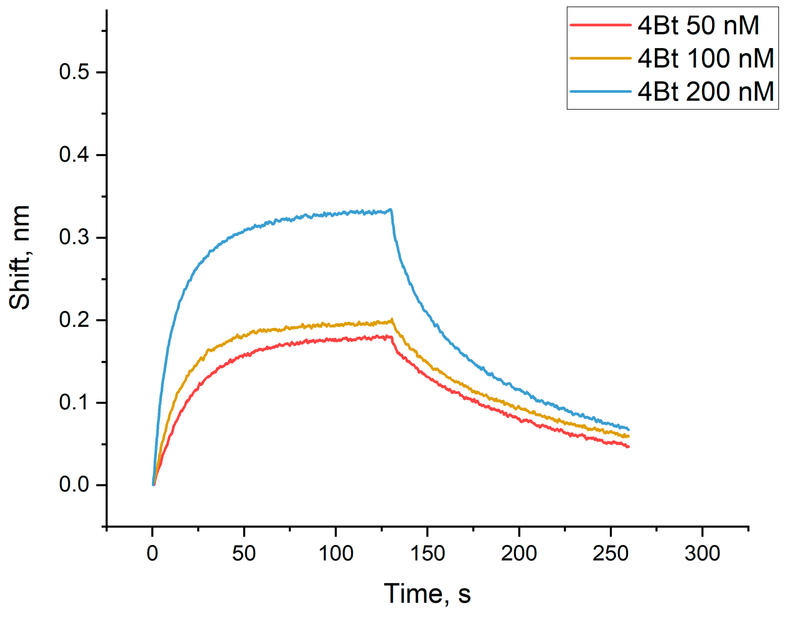

Figure S21. Binding curves of RBD expressed in CHO cell line to immobilized aptamer CoV2-RBD-4-Biotin.

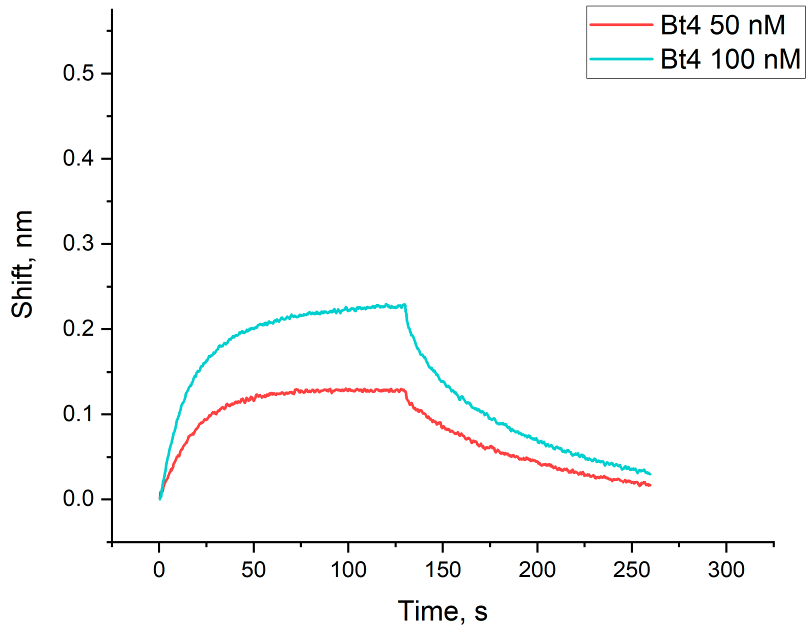

Figure S22. Binding curves of RBD expressed in CHO cell line to immobilized aptamer Biotin-CoV2-RBD-4.

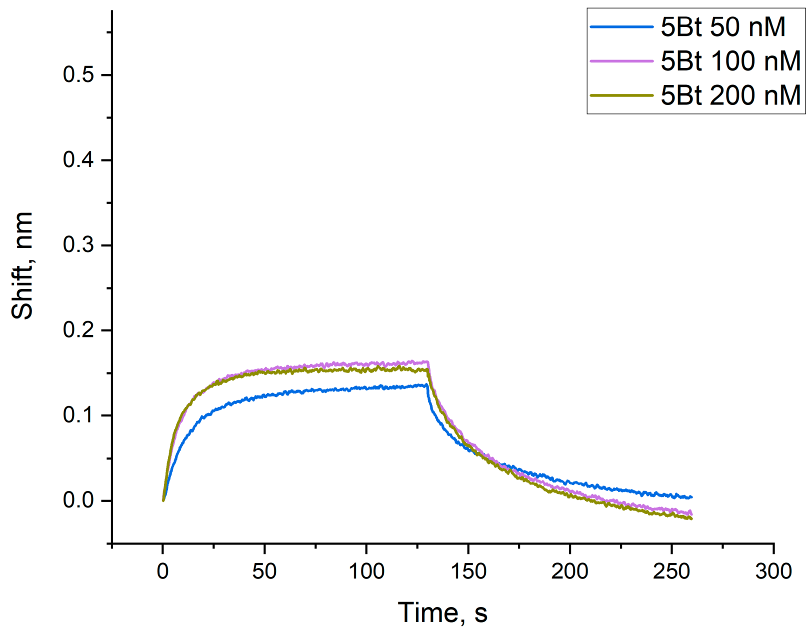

Figure S23. Binding curves of RBD expressed in CHO cell line to immobilized aptamer CoV2-RBD-5-Biotin.

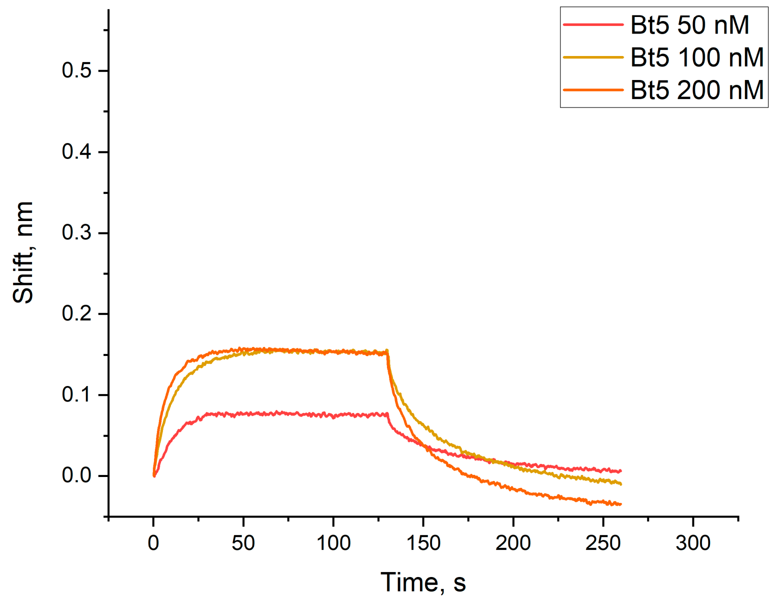

Figure S24. Binding curves of RBD expressed in CHO cell line to immobilized aptamer Biotin-CoV2-RBD-5.

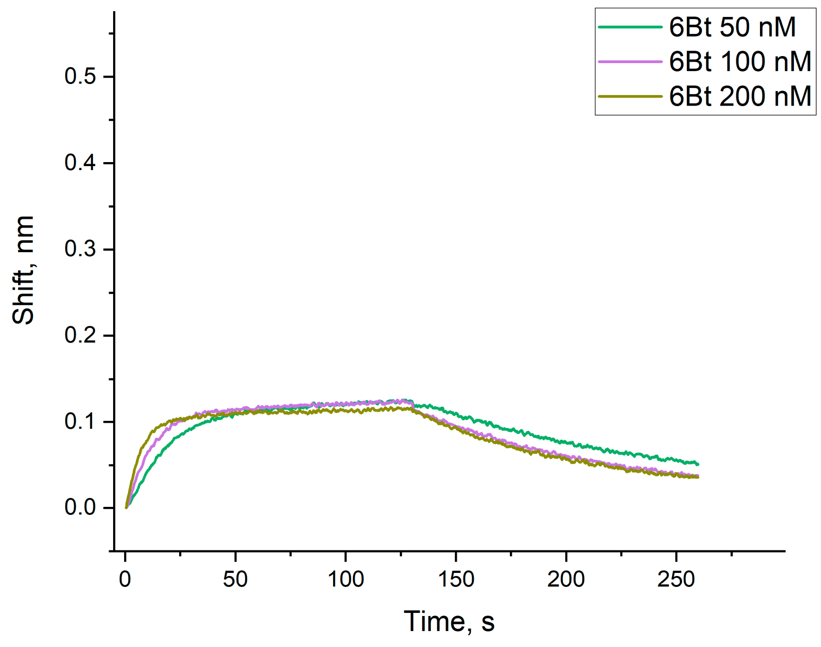

Figure S25. Binding curves of RBD expressed in CHO cell line to immobilized aptamer CoV2-RBD-6-Biotin.

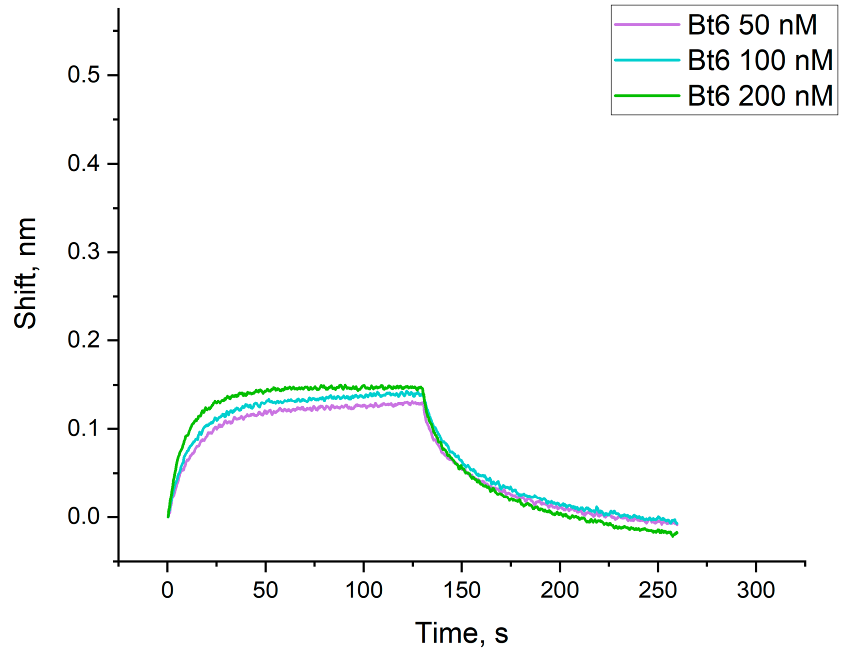

Figure S26. Binding curves of RBD expressed in CHO cell line to immobilized aptamer Biotin-CoV2-RBD-6.
